# The Waiting Time for Inter-Country Spread of Pandemic Influenza

**DOI:** 10.1371/journal.pone.0000143

**Published:** 2007-01-03

**Authors:** Peter Caley, Niels G. Becker, David J. Philp

**Affiliations:** National Centre for Epidemiology and Population Health, Australian National University, Canberra, Australia; University of Ioannina School of Medicine, Greece

## Abstract

**Background:**

The time delay between the start of an influenza pandemic and its subsequent initiation in other countries is highly relevant to preparedness planning. We quantify the distribution of this random time in terms of the separate components of this delay, and assess how the delay may be extended by non-pharmaceutical interventions.

**Methods and Findings:**

The model constructed for this time delay accounts for: (i) epidemic growth in the source region, (ii) the delay until an infected individual from the source region seeks to travel to an at-risk country, (iii) the chance that infected travelers are detected by screening at exit and entry borders, (iv) the possibility of in-flight transmission, (v) the chance that an infected arrival might not initiate an epidemic, and (vi) the delay until infection in the at-risk country gathers momentum. Efforts that reduce the disease reproduction number in the source region below two and severe travel restrictions are most effective for delaying a local epidemic, and under favourable circumstances, could add several months to the delay. On the other hand, the model predicts that border screening for symptomatic infection, wearing a protective mask during travel, promoting early presentation of cases arising among arriving passengers and moderate reduction in travel volumes increase the delay only by a matter of days or weeks. Elevated in-flight transmission reduces the delay only minimally.

**Conclusions:**

The delay until an epidemic of pandemic strain influenza is imported into an at-risk country is largely determined by the course of the epidemic in the source region and the number of travelers attempting to enter the at-risk country, and is little affected by non-pharmaceutical interventions targeting these travelers. Short of preventing international travel altogether, eradicating a nascent pandemic in the source region appears to be the only reliable method of preventing country-to-country spread of a pandemic strain of influenza.

## Introduction

The emergence of a pandemic strain of influenza is considered inevitable [1]. Provided the emerged strain is not too virulent, it may be possible to eliminate a nascent influenza pandemic in the source region via various combinations of targeted antiviral prophylaxis, pre-vaccination, social distancing and quarantine [2], [3]. If early elimination in the source region is not achieved, then any delay in a local epidemic that a country can effect will be highly valued. To this end, countries may consider introducing non-pharmaceutical interventions such as border screening, promoting early presentation of cases among arriving passengers, requiring the use of personal protective equipment during travels (e.g. the wearing of masks), and reducing traveler numbers. While the case for believing that measures such as these can not stop the importation of an epidemic from overseas has been argued strongly, whether it be SARS or influenza [4]–[6], the extent to which such interventions delay a local epidemic is currently not well quantified, and hence of considerable interest.

In this paper we demonstrate how the delay to importation of an epidemic of pandemic strain influenza may be quantified in terms of the growing infection incidence in the source region, traveler volumes, border screening measures, travel duration, in-flight transmission and the delay until an infected arrival initiates a chain of transmission that gathers momentum. We also investigate how the delay is affected by the reproduction number of the emerged strain, early presentation of cases among arriving passengers, and reducing traveler numbers. As noted in previous simulation modeling [7], many aspects of this delay have a significant chance component, making the delay a random variable. Therefore, the way to quantify the delay is to specify its probability distribution, which we call the delay-distribution.

Some issues of the delay distribution, such as the natural delay arising in the absence of intervention and the effect that reducing traveler numbers has on this delay has been studied previously [6]–[8]. Specifically, if the originating source is not specified, and homogeneous mixing of the worlds population is assumed, then the most likely time to the initial cases arising in the United States is about 50 days assuming *R*_0_ = 2.0 [7]. The additional delay arising from travel restrictions appears minimal until a>99% reduction in traveler numbers [6]–[8].

This paper adds to previous work [5]–[8] by simultaneously including a wider range of epidemiological factors and possible interventions, such as elevated in-flight transmission, flight duration, the effect of wearing of mask during flight, early presentation of cases among travelers, and quarantining all passengers from a flight with a detected case at arrival.

## Methods

### General

Consider a region in which a new pandemic strain of influenza has emerged, and a region currently free from the infection. We refer to these as the *source region* and the *at-risk country*, respectively. Travel between these countries is predominantly via commercial air travel and/or rapid transport which could potentially be subject to border screening and other interventions. We restrict our discussion to air travel. The aim is to assess the effects that a variety of non-pharmaceutical border control measures have, individually and in combination, on the time it takes before the epidemic takes off in the at-risk country. An epidemic is said to have “taken off” when it reaches 20 current infectious cases, after which its growth is highly predictable (i.e. nearly deterministic) and the probability of fade-out by chance is very low, if intervention is not enhanced. The source country of origin will undoubtedly have a large impact on the natural delay until importation of an epidemic, although this is difficult to quantify [7]. An alternative is to fix the originating city, for example a highly connected city such as Hong Kong [6], with the obvious effect that results are highly dependent on the choice. We adopt no specific source region, but assume that the number of international travelers originating from it is reasonably small (see Methods), suggestive of a rural or semi-rural source region [2]. It is further assumed that the current heightened surveillance for pandemic influenza is continued and that a nascent pandemic with human-to-human transmission is identified and the pandemic is declared when there are 10 concurrent cases in the source region.

For an epidemic to take off in an at-risk country, a series of events need to occur. First, the epidemic needs to get underway in the source region. Second, an intending traveler needs to be infected shortly before departure. Third, the infected traveler must actually travel and successfully disembark in the at-risk country. Fourth, the infected traveler, or fellow travelers infected during the flight, must initiate an epidemic in the at-risk country with the infectiousness that remains upon arrival. Finally, the epidemic needs to reach a sufficient number of cases to begin predictable exponential growth.

### Infected travelers

**Figure 1:**
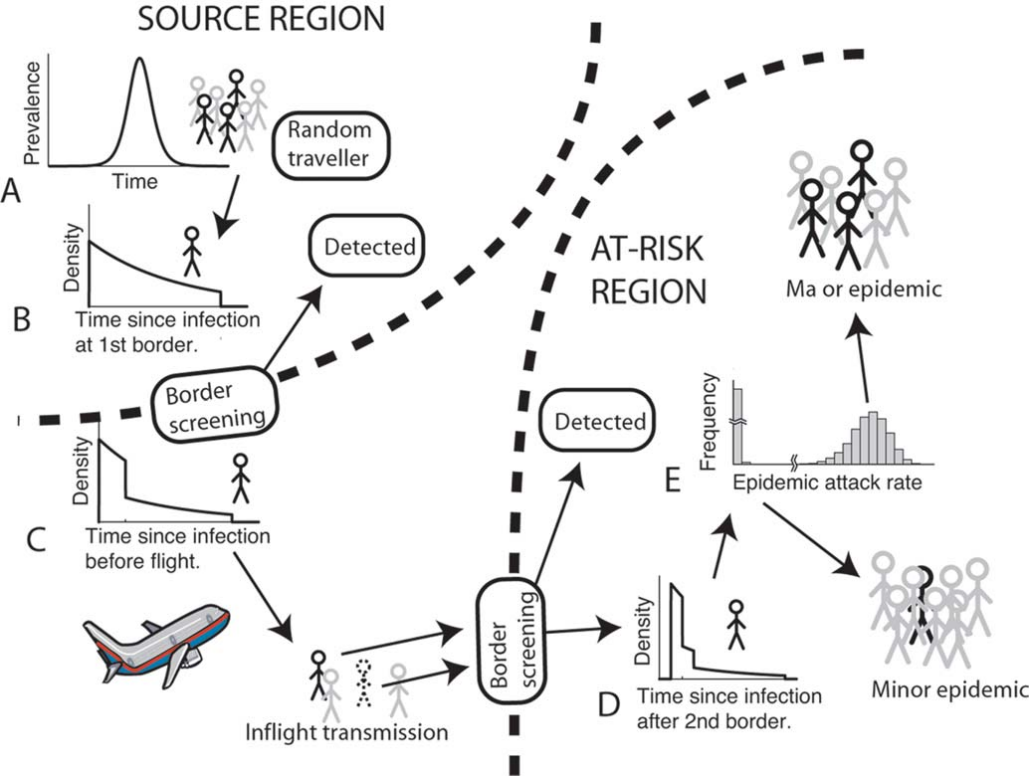
The process through which a pandemic is imported. (A) The prevalence in the source region, which determines the probability that a randomly selected traveler is infected at scheduled departure. (B)–(D) Density functions of the time since infection during the early stages of the epidemic in the source region for infected travelers (B) before and (C) after departure screening, and (D) after arrival screening for clinical symptoms. In (B), the step illustrates the probabilistic removal of travelers who have completed their incubation period. In (D), the distribution of time since infection in (C) will have shifted to the right by an amount equal to the flight duration, and cases incubated in-flight may be detected by symptomatic screening, as will those symptomatic cases that were not detected previously. Screening sensitivity for this illustration is 60% on both departure and arrival. (E) Upon entering the community undetected, an infected traveler may initiate a minor (inconsequential) or major epidemic, depending on the characteristics of the disease and public health policy.

International spread of the emerged pandemic strain of influenza may occur when a recently infected person travels. By ‘recently infected’ we mean that their travel is scheduled to occur within ten days of being infected. We assume that the number of individuals traveling from the source region to the at-risk country each day is known. The probability that a randomly selected traveler is a recently-infected person is taken to be equal to the prevalence of recently-infected people in the source region on that day. The incidence of infection in the source region is assumed to grow exponentially initially, with the rate of exponential growth determined by the disease reproduction number (the mean number of cases a single infective generates by direct contact) and the serial interval (the average interval from infection of one individual to when their contacts are infected) (Figure 1A).

The time since infection of a recently-infected traveler is a key component of the calculations, because it affects the chance of positive border screening, the chance of in-flight transmission and the infectivity remaining upon arrival in the at-risk country. The time since infection at the time of scheduled departure is random and the dependence of its probability distribution on the exponential growth rate of infection is illustrated by Figure 1B (see also Supporting Information). The higher the epidemic growth rate in the source region, the greater the probability than an infected traveler will have been infected more recently.

### Traveler screening at departure

It is assumed that individuals detected by departure screening are prevented from traveling. To be detected by screening an infected traveler must be symptomatic and positively screened. An individual is assumed to become symptomatic 48 hours after being infected (cf. [3] who use 1.9 days). The probability of being symptomatic when presenting for departure screening is computed from the curve in Figure 1B. The distribution of the time since infection immediately after departure screening, given that the infected traveler was not detected, is given by the curve in Figure 1C. It contains an adjustment for the probability of being detected at departure.

### In-flight transmission

The instantaneous rate at which susceptible contacts are infected depends on the time since infection, and is described by an infectiousness function ([9], page 45). We use a peaked infectiousness function, motivated by viral shedding and household transmission data [2], which has a serial interval of 2.6 days. The basic reproduction number (*R*_0_), namely the reproduction number when there is no intervention in place and every contacted individual is susceptible, is given by the area under the infectiousness function. However, our concern is with the effective reproduction number *R* that holds when various interventions are in place. We obtain any *R* by simply multiplying the infectiousness function by the appropriate constant (to make the area under the curve equal to *R*). This keeps the serial interval the same. In the absence of suitable data we assume for most scenarios that the aircrafts ventilation and filtration systems are functioning properly, and that infected travelers transmit the infection at the same rate during a flight as they would while mixing in the community. We examine the sensitivity of this assumption by increasing the in-flight transmission by as much as 10-fold (as could potentially happen if air-circulation and filtration systems malfunction, e.g. see [10]). The in-flight transmission rate is set to zero under the optimistic scenario that all travelers wear 100% effective masks during transit. In terms of a sensitivity analysis this illustrates what would be achievable in a best-case scenario. The number of offspring that an infected traveler infects during a flight is a random variable, taken to have a Poisson distribution with a mean equal to the area under the infectiousness function over to the flight duration.

### Traveler screening at arrival

Travelers infected during flights of less than 12 hours duration are asymptomatic at arrival and will not be detected by screening. The probability that an arriving traveler who was infected in the source region is detected on arrival is computed from the distribution of the time since infection on arrival. This distribution is obtained from the curve in Figure 1C by shifting it to the right by an amount equal to the duration of the flight. The distribution of the time since infection for an individual infected in the source region, who passes through arrival screening undetected has a further adjustment for the chance of being detected at arrival (Figure 1D). This curve shows that an infected traveler who escapes detection at departure and arrival is highly likely to enter the at-risk country with most, or all, of their infectious period remaining.

Authorities are assumed to implement one of two control options when detecting an infected traveler by arrival screening. Under option one (individual-based removal), all passengers who test negative are released immediately and only passengers who test positive are isolated. Under the second option (flight-based quarantining), authorities prevent all passengers from dispersing into the community until the last person has been screened from that flight. Should any one passenger be detected as infected then all passengers will be quarantined, as previously recommended [5].

### Transmission chains initiated by infected arrivals

Transmission chains can be initiated in the at-risk country by infected travelers who mix within the community upon arrival. Suppose now that a flight arrives with one, or more, infected passengers who mix within the community. We classify these infected arrivals into those who are ‘pre-symptomatic’ and those who are ‘symptomatic’ at entry. It is assumed that the ‘symptomatic’ infected arrivals do not recognize their symptoms as pandemic influenza and will not present to medical authorities. In other words, they spend the remainder of their infectious period mixing in the community. On the other hand, the ‘pre-symptomatic’ infected arrivals, including all individuals infected during flight, are assumed to mix freely in the community only from entry until they present to medical authorities after some delay following the onset of symptoms.

### Probability that an undetected infected traveler initiates a major epidemic

Not all infected travelers entering the community initiate a ‘major’ epidemic, even when the reproduction number (*R*) exceeds one. Quite generally, the distribution of the size of an epidemic initiated by an infected arrival is bimodal, with distinct peaks corresponding to a major epidemic and a minor outbreak (Figure 1E). In the latter event the outbreak simply fades out by chance despite there being ample susceptibles in the population for ongoing transmission [11]. The number of cases in an outbreak that fades out is typically very small compared to an epidemic.

The probability that a typical infective generates a local epidemic is computed by using a branching process approximation [12] for the initial stages of the epidemic, and equating ‘epidemic’ with the event that the branching process does not become extinct. This calculation is well known (e.g. [13], page 473), but is modified here to allow for the fact that the process is initiated by a random number of infected arrivals and some of them have spent a random part of their infectious period before arriving in the at-risk country. The distribution for the random number of individuals infected by an infected individual when all their contacts are with susceptible individuals is needed for the calculation. The lack of data prevents a definitive conclusion for the most appropriate offspring distribution for influenza transmission [14], and we use a Poisson distribution with a mean equal to *R*, discounted for individuals who spent only some of their infectious period mixing in the at-risk country. A Poisson offspring distribution is appropriate when the area under the infectiousness function is non-random (i.e. all individuals have the same infection ‘potential’). We assume that *R* is the same in the source region and the at-risk country. For an undetected infected traveler and all their in-flight offspring to fail to initiate an epidemic on arrival, all of the chains of transmission they initiate must fail to become large epidemics (see Supporting Information).

### The delay until an epidemic gathers momentum in the at-risk country

We calculate the probability distribution of *D*, the total delay until an epidemic gathers momentum by noting that it is given by *D* = *D*_1_+*D*_2_, where *D*_1_ is the time until an epidemic is first initiated and *D*_2_ is the time from initiation until the local epidemic gathers momentum. For an epidemic to be first initiated in the at-risk country on day *d*, it must have not been initiated on all previous days. Hence the probability distribution of the time delay (*D*_1_) until the epidemic is first initiated in the at-risk country following identification in the source region is described by:
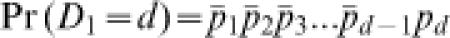
where *p_d_* denotes the probability that the epidemic is initiated on day *d* , and 
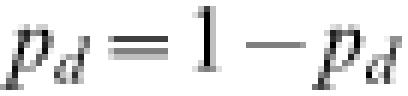
 denotes the probability that the epidemic is *not* initiated on day *d* (see Supporting Information for calculation of *p_d_*).

Once successfully initiated, an epidemic may initially hover around a handful of cases before reaching a sufficient number of cases for its growth to become essentially predictable. As mentioned, 20 concurrent cases is our criterion for an epidemic to have gathered momentum. We determine the distribution of *D*_2_, the time to this occurrence, from 10,000 stochastic simulations and approximate this empirical distribution by a shifted gamma distribution. Our criterion of 20 concurrent cases is conservatively high, as results from the theory of branching processes shows that the probability of a minor epidemic (and hence no take-off) starting from 20 concurrent cases is about 3×10^−8^ when *R* = 1.5, and even smaller for higher values of *R*. Finally, the distribution of the total delay (*D* = *D*_1_+*D*_2_) from the pandemic being identified in the source region until 20 cases in the at-risk country was calculated by the convolution of the distributions of *D*_1_ and *D*_2_.

### Parameter values

For the illustrative purposes, we chose values of 1.5, 2.5 and 3.5 for *R*, which encompass estimates proposed for previous pandemics [2], [3], [15]. The number of people within the infected source region was assumed reasonably small (5 million), and there was one flight per day traveling from the source region to the at-risk country carrying 400, 100 or 10 passengers. A higher number of travelers affects the delay only marginally, assuming the epidemic takes off in the source region (see Results). We assume a typical travel duration between attempted departure and possible arrival of 12 hours, but also examine the effect of varying this from 0–48 hours. The time to presentation following symptom onset is varied from ‘immediately’ to ‘never presenting’, with a time of 6 hours considered likely in the presence of an education campaign. The sensitivity of symptomatic screening is varied from 0–100%, with results presented for 0, 50 and 100% sensitivity.

## Results

### Evading traveler screening

**Figure 2:**
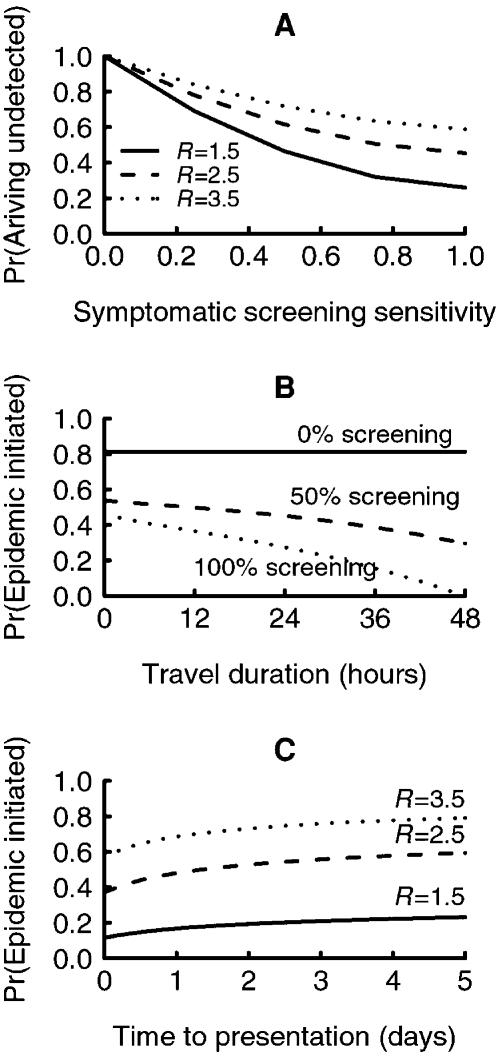
Effects of border screening and early presentation. (A) The effects of screening sensitivity andon the probability of escaping detection on both departure and arrival during a 12 hour transit. (B) The effects of screening sensitivity and travel duration on the probability than an infected traveler escapes detection during transit and initiates an epidemic after arrival (assuming no other symptomatic individuals on the same flight are identified). *R* = 3.5 with no early presentation. (C) The effects of *R* and the time from symptom onset to presentation on the probability that an infected traveler, having entered the wider community following arrival, will initiate an epidemic. There is no screening.

The probability that a recently infected traveler evades screening is substantial even if screening reliably detects symptomatic travelers (Figure 2A), because the typical travel duration is shorter than the 2-day incubation period. In addition, during the early stages of the epidemic a high *R* in the source region acts to increase the probability that an infected traveler has been infected quite recently and hence will escape detection due to being asymptomatic during their travels (Figure 2A). For example, assuming 100% sensitivity for detecting symptomatic infection, we calculate that during the early stages of the epidemic the proportion of infected travelers that evade both departure and arrival screening after 12 hours of travel is 0.26, 0.45 and 0.59 for disease reproduction numbers 1.5, 2.5 and 3.5, respectively.

As the duration of travel approaches the disease incubation period, effective symptomatic screening substantially reduces the likelihood that a traveler evades screening and initiates an epidemic (Figure 2B). Reducing the time from the onset of symptoms to presentation (and subsequent isolation) for each infected arrival also reduces the probability that a major epidemic is initiated, however the best case scenario of infected travelers and all their in-flight offspring presenting immediately following the onset of symptoms still poses a substantial risk of epidemic initiation arising from pre-symptomatic transmission (Figure 1C).

### The time until an epidemic gathers momentum in the at-risk country

**Figure 3:**
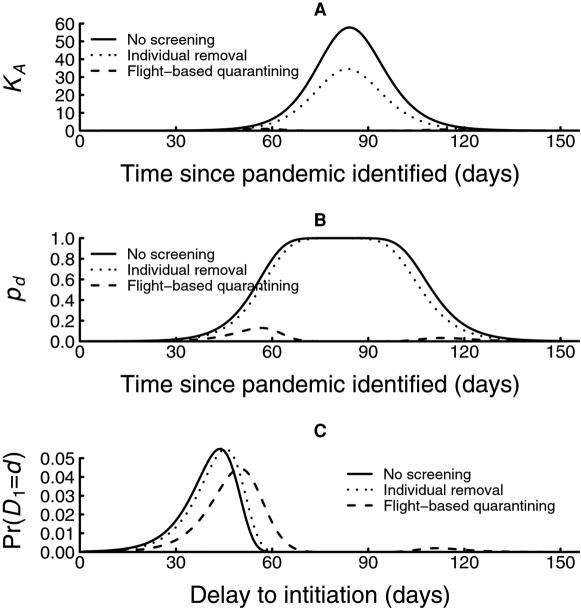
Components of delay until initiation and effects of border screening. (A) The number of infected people successfully arriving and entering the community of an at-risk country (*K_A_*) on each day following the identification of an outbreak of pandemic type strain influenza, assuming a source region population of 5 million, 400 intending travelers per day, *R* = 1.5, and three levels of symptomatic screening (solid line = nil, dashed line = 50% sensitivity with individual-based removal, dotted line = 50% sensitivity with flight-based quarantining). (B) Corresponding daily probability of initiation (*p_d_*) as a function of time since pandemic identified. (C) Distribution of the delay time until the initiation (*D*_1_) of an epidemic in an at-risk country by an infected traveler from a source region.

**Figure 4:**
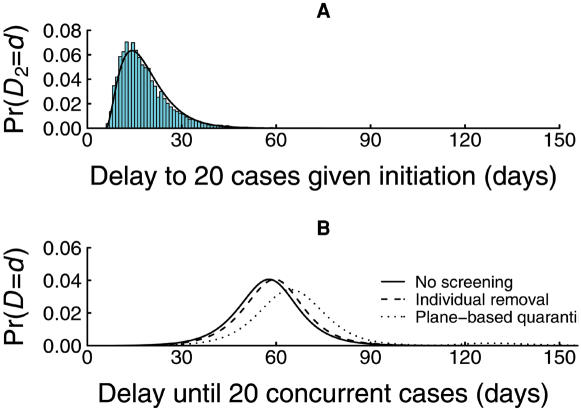
Components of the delay in at-risk country following initiation. (A) Results of 10,000 simulations (bars) and fitted shifted-Gamma distribution of delay time (*D*_2_) until 20 concurrent cases occur in the at-risk country, given that an epidemic has been initiated, andequals 1.5 with a serial interval of 2.6 days. (B) The total delay distribution until there are 20 concurrent cases in the at-risk country from when a pandemic type strain of influenza outbreak is identified in a source region with a population of 5 million, 400 intending travelers day^−1^, an R of 1.5, and three levels of symptomatic screening (solid line = nil, dashed line = 50% sensitivity with individual removal, dotted line = 50% sensitivity with flight-based quarantining).

The delay contains a fairly substantial *natural* component, primarily due to the time it takes to increase the number of infectives in the source region sufficiently to make the chance of a recently infected traveler appreciable (Figure 3A), and the time (*D*_2_) it takes for a local epidemic in the at-risk country to gather momentum following successful seeding (Figure 4A). In the absence of any interventions, the number of infected individuals who successfully enter the community of the at-risk country initially increases exponentially (Figure 3A). With individual-based removal of infected travelers, the number of individuals entering the at-risk country undetected by screening is proportionately reduced over the course of the epidemic (Figure 3A). With flight-based quarantining, the number of infected individuals entering the at-risk country undetected is dramatically reduced over the course of the epidemic, even for relatively insensitive screening (Figure 3A). With flight-based quarantining, the number of infected passengers slipping through undetected is bimodal, with the first peak occurring when the number of infected travelers attempting to travel is still in single figures.

Without screening, the daily probability that an epidemic is initiated (*p_d_*) increases, and becomes near certain once the number of infected travelers arriving undetected exceeds about 10 (Figure 3B, solid line). With screening and individual-based removal of infected individuals, *p_d_* follows a similar pattern only reduced somewhat. With screening in combination with flight-based quarantining, this probability is changed dramatically. After an initial rise it dips, to become essentially zero during the height of the epidemic in the source region (Figure 3B, dotted line). This arises because once a flight has several infected travelers, the probability that at least one is detected approaches one (even if screening is imperfect), and all passengers on such a flight are quarantined. Once the epidemic starts to wane in the source region (assuming the unlikely event of the pandemic strain is restricted to the source region), the probability of initiation rises once again. The corresponding distribution of *D*_1_, the delay until the epidemic is first initiated in the at-risk country, is bi-modal in the presence of screening (Figure 3C).

Although flight-based quarantining is effective in preventing the entry of infected travelers during the height of the epidemic, a substantial cumulative risk of initiation has already occurred before this from the handful of infectives that have slipped through undetected (Figure 3B). Hence, whilst the effect of border screening, particularly in conjunction with flight-based quarantining, on the daily probability of initiation is dramatic, its effect on the delay to initiation is much less pronounced (Figure 3C). Border screening, even with perfect sensitivity for detecting symptomatic cases, tends to increase *D*_1_, the time to an epidemic being initiated, by a matter of days to weeks. The time (*D*_2_) from initiation (the arrival of the index case) to an epidemic reaching 20 concurrent cases within the at-risk country is adequately modeled using a shifted Gamma distribution (Figure 4A). The convolution of this right-skewed Gamma distribution with the left-skewed delay-distribution of *D*_1_ (Figure 3C) yields the distribution for *D*, the total delay until the epidemic reaches 20 cases in the at-risk country (Figure 4B). The distribution of *D* is approximately symmetrical. The effect of border screening on the total delay *D* is quite modest, though sensitive to how screening is implemented. For example, with *R* = 1.5 and 400 travelers per day, 100% sensitive screening with individual-based removal increases the median delay from 57 to 60 days (Figure 4B). Flight-based quarantining would extend the median delay to 70 days. In general, the added delay arising from flight-based quarantining is about four-fold that arising from individual-based removal.

**Figure 5:**
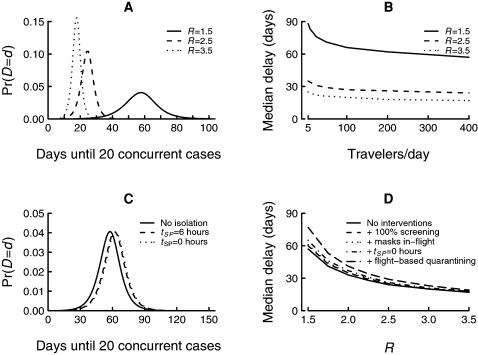
Effects of interventions on the total delay *D*. (A) The effects of *R* on delay-distribution. (B) The effects of daily traveler number on the median delay for different values of *R*. (C) The effects of the time from symptom incubation until presentation and isolation (*t_SP_*) on the delay-distribution. (D) Additive effects of implementing 100% sensitive border screening (individual removal), the wearing of masks during transit, immediate presentation following symptom onset, and flight-based quarantining on the median delay, assuming 400 travelers per day attempting to depart the source region.

The natural component of the delay is highly sensitive to the disease reproduction number (Figure 5A). For example, with 400 passengers per day departing the source country and in the absence of any interventions, the median delay ranges from a low of 17 days for *R* = 3.5 to 57 days for *R* = 1.5 (Table 1). The delay is less sensitive to the number of intending travelers, with little appreciable increase in the median delay occurring until traveler numbers become very low (Figure 5B). For example, if *R* = 1.5, with no other border control measures, decreasing the number of intending travelers departing the source region from 400 to 100 per day increases the median total delay *D* from 57 to 66 days. A further decrease in the number of intending travelers to 10 per day increases the median delay to 83 days (Table 1).

**Table 1:**
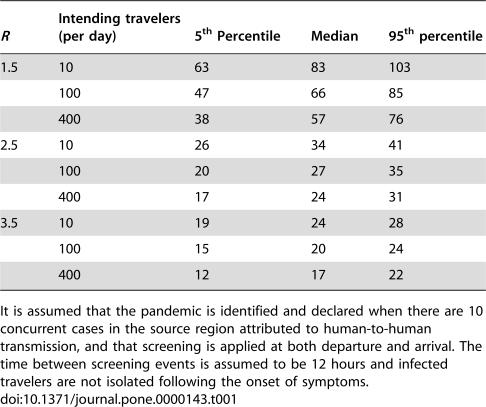
Summary measures of the expected time until an epidemic of pandemic strain influenza in an at-risk country reaches 20 cases, for three values of *R* and three values for the number of intending travelers when the source region contains 5 million people.

*R*	Intending travelers (per day)	5^th^ Percentile	Median	95^th^ percentile
1.5	10	63	83	103
	100	47	66	85
	400	38	57	76
2.5	10	26	34	41
	100	20	27	35
	400	17	24	31
3.5	10	19	24	28
	100	15	20	24
	400	12	17	22

It is assumed that the pandemic is identified and declared when there are 10 concurrent cases in the source region attributed to human-to-human transmission, and that screening is applied at both departure and arrival. The time between screening events is assumed to be 12 hours and infected travelers are not isolated following the onset of symptoms.

The delay is quite insensitive to the rate of transmission in-flight. For example, with *R* = 1.5, a 12-hour flight, 400 travelers per day and no other interventions, preventing in-flight transmission altogether increases the median delay from 57 to 58 days. Conversely, doubling the rate of in-flight transmission reduces the median delay from 57 to 56 days. A 10-fold increase in the rate of transmission in-flight only decreases the median delay from 57 to 53 days. Encouraging the early presentation of cases among travelers following the onset of symptoms has a limited effect on the delay distribution (Figure 5C). For example, for *R* = 1.5, 400 intending travelers per day and no other interventions, reducing the time to presentation from ‘never presenting’ to 6 hours increases the median delay from 57 to 61 days. Immediate presentation at symptom onset only increases the median delay a further day in this scenario.

In general, the additional delay achieved by introducing non-pharmaceutical border control measures is generally small in comparison with the natural delay (Figure 5D). For the scenario with *R* = 1.5 and 400 intending travelers per day, a combination of 100% flight-based quarantining, 100% compliance with mask wearing during travel and immediate presentation at symptom onset extends the estimated median delay from 57 to 79 days (Figure 5D). This added delay diminishes in absolute terms as *R* increases. For example, if the same interventions are applied with *R* = 3.5, the median delay is extended from 17 to just 20 days (Figure 5D). The one exception to this generalisation is when travel numbers are reduced dramatically. The added delay achieved when a drastic reduction in travel numbers is combined with other border control measures appears to be greater than adding the delays each achieves on its own. For example, if *R* = 1.5, and we reduce the number of intending travelers from 400 to 10 per day, implement 100% flight-based quarantining, implement compulsory mask wearing during travel and presentation at 6 hours following symptom onset then there is a substantial probability (0.74) that the pandemic strain will never be imported (assuming the epidemic is confined to the source country). The estimated quartile delay (the median in this case is undefined) to the start of a major epidemic in an at-risk country is extended from 50 to 125 days. Again, the added delay decreases rapidly as *R* increases, and if the above interventions were applied with *R* = 3.5, the estimated median delay is extended from 17 to 26 days, and the importation of the epidemic is certain (Figure 5D).

## Discussion

We have formulated a model of the importation of an infectious disease from a source region to an at-risk country that permits a comprehensive analysis of the effect of border control measures. Our results are most relevant to the early stage of a pandemic when most cases are contained within a single source region. Once the pandemic has spread to several countries, models with greater complexity and ability to more realistically model global mixing patterns [6]–[8] are required. Our model is developed with a pandemic-strain of influenza in mind, but could apply to any emerging infectious disease that is transmitted from person to person. We have assumed a Poisson distribution for the number of secondary infections, which a natural choice when each infected individual has the same infectivity profile. A distribution with a larger variance is appropriate when individuals vary substantially in their infectiousness. Our results are conservative in the sense that they give an upper bound for the probability that an infected traveler manages to initiate an epidemic, compared to an offspring distribution with a greater variance but the same reproduction number [14].

The nature of the next pandemic influenza virus, and particularly its reproduction number, is uncertain. If its reproduction number is low (*R*<2.0), our results indicate that at-risk countries receiving a reasonably small number of travelers (say 400 per day) from the infected source region can expect a natural delay until importing an epidemic of the order of 2 months. This is quite variable and under favourable conditions it could be 4 months. However, the natural delay decreases rapidly as *R* increases.

The additional delay from isolating individuals detected by border screening is merely a few days under most plausible scenarios, even if both departure and arrival screening is introduced and screening detects every symptomatic traveler. While the extra delay is more than quadrupled if flights with a detected case(s) are quarantined, the effect remains modest (weeks at most) and it is questionable whether the extra delay achieved warrants the disruption created by such a large number of quarantined passengers.

In-flight transmission is a commonly raised concern in discussions about the importation of an infection, so inclusion of in-flight transmission is an attractive feature of our model. Events of substantial in-flight transmission of influenza have been documented [10], [16] and modeling of indoor airborne infection risks in the absence of air filtration predicts that in-flight transmission risks are elevated [17]. However, it difficult to estimate the infectiousness of influenza in a confined cabin space, as there is undoubtedly substantial under-reporting of influenza cases who travel and fail to generate any offspring during flight. Provided the aircraft ventilation system (including filtration) is operational, it is considered that the actual risk of in-flight transmission is much lower than the perceived risk [18]. Our results indicate that the delay is relatively insensitive to the rate of in-flight transmission, making in-flight transmission less of an issue than commonly believed. A highly elevated transmission rate in-flight will hasten the importation of an epidemic only marginally. Consistent with this, eliminating in-flight transmission by wearing protective masks increases the delay only marginally.

Early presentation by infected arrivals not detected at the borders was found to add only a few days to the delay. To some extent this arises due to our assumption that pre-symptomatic transmission can occur, for which there is some evidence. In contrast, Ferguson *et al*. [2] assume that the incubation and latent periods are equal, with a mean of 1.5 days. In their model pre-symptomatic transmission is excluded and infectiousness is estimated to spike dramatically immediately following symptom onset and declining rapidly soon afterwards. Under their model assumptions, immediate presentation at onset of symptoms would reduce transmission effectively. However, as presentation occurs some time after onset of symptoms and the bulk of infectivity occurs immediately after onset of symptoms the results on the effect of early presentation of cases are likely, in practical terms, to be similar to those found here. Given the variable nature of influenza symptoms, there is likely to be a difference between the onset of the first symptoms as measured in a clinical trial (e.g. [19]) and the time that a person in the field first suspects that they may be infected with influenza virus. To fully resolve the issue of how effective very early presentation of infected travelers is in delaying a local epidemic we need better knowledge about the infectiousness of individuals before and just after the onset of symptoms.

Of the border control measures available, reducing traveler numbers has the biggest effect on the delay and even then it is necessary to get the number of travelers down to a very low number. An equivalent control measure is to quarantine all arriving passengers with near perfect compliance.

Our results indicate that short of virtually eliminating international travel, border control measures add little to avoiding, or delaying, a local epidemic if an influenza pandemic takes off in a source region. All forms of border control are eventually overwhelmed by the cumulative number of infected travelers that attempt to enter the country. The only way to prevent a local epidemic is to rapidly implement local control measures that bring the effective reproduction number in the local area down below 1, or to achieve rapid elimination in the source region, in agreement with other recent studies [6]–[8]. Preventing the exponential growth phase of an epidemic in the source region appears to be the only method able to prevent a nascent influenza pandemic reaching at-risk countries.

## Supporting Information

Text S1 Estimating the daily probability of epidemic initiation
(0.08 MB PDF)
