# Supplementary material for: The Waiting Time for Inter-Country Spread of Pandemic Influenza
Source: PLoS One. 2007 Jan 3;2(1):e143. doi: 10.1371/journal.pone.0000143 (PMC1764036; doi:10.1371/journal.pone.0000143)
Supplement: Text S1 — Estimating the daily probability of epidemic initiation (0.08 MB PDF) [file pone.0000143.s001.pdf]

Estimating the daily probability of epidemic initiation

Peter Caley, Niels G. Becker and David J. Philp

December 14, 2006

Specifying distribution of time since infection

For each day of the epidemic in the source region, the exponential rate of increase (ρ) of the epidemic was estimated from the solution of Lotka's equation:

$$s \int_0^\infty e^{-\rho u} \beta(u) du = 1,$$

where s is the proportion of susceptibles in the population and $\beta(x)$ is the infectiousness function. Note that the area under the infectiousness function equals R. That is, $\int_{t=0}^{t=\infty} \beta(u) du = R$. If the number of infectious cases is changing at exponential rate ρ, it follows that the probability distribution of the time since infection of a randomly selected infective has density function $f_T(t) = a \exp(-\rho t)$, where a is an appropriate constant (proof omitted).

Offspring generated in at-risk country community from infected traveler and their in-flight offspring

We assume that the number of offspring generated during the flight (X) is distributed as Poisson with rate parameter equal to the area under the time-dependent infectiousness function corresponding to the flight duration (d_F). Hence, conditional on the time since infection upon departure of the infected traveler, the number of offspring they generate in-flight is

$$X \Bigm| T = t \;\sim\; \text{Pois}\left(\lambda \int_t^{t+d_F} \beta(u) du\right),$$

where λ is a scalar which alters the rate of transmission in-flight (e.g. $\lambda = 0$ if all passengers wear masks).

In-flight offspring will have virtually no chance of becoming symptomatic before arrival and hence being detected by symptomatic border screening. Upon entering the community of the at-risk country, the X in-flight offspring will infect $Z_1 + Z_2 + \cdots + Z_X$ offspring

in the wider community. The Z_i are independent and identically distributed as Poisson with mean R (the effective reproduction number within the wider community). For the index infected traveler whose infection originated in the source country and who enters the community, the number of offspring they generate (Y) is distributed as Poisson with its mean determined by the infectious contacts made from the time of disembarking until presentation to medical authorities after some delay (δ) following the onset of symptoms, conditional on the time since infection upon departure. That is,

$$Y \Big| T = t \;\sim\; \text{Pois}\left(\int_{t+d_F}^{t+d_F+\delta} \beta(u)du \right).$$

In total, an infected traveler generates N_0 chains of infection in the at-risk country , where

$$N_0 \;=\; Y + \sum_{i=1}^{X} Z_i \,.$$

Calculating the daily probability of minor epidemic arising from an infected traveler and their in-flight offspring in presence of individual removal

Branching models enable calculation of the probability that a minor or major epidemic results from the event that an infected traveler and/or their inflight offspring enter the community. Let q be the probability that a newly infected individual introduced to a fully susceptible community fails to initiate a major epidemic. Assuming that the offspring distribution is distributed as Poisson with rate parameter R, this probability is specified by the smaller solution of the equation $q = \exp(-R(1-q))$. We want to estimate the overall probability q_0 of a minor epidemic arising from all epidemic chains initiated by the infected traveler and their in-flight offspring, where the probability of any one initiated chain resulting in a minor epidemic equals q. For an infected traveler and all their in-flight offspring to fail to initiate an epidemic on arrival, all N_0 chains must fail to become large epidemics. That is, the probability of a minor epidemic arising from chains initiated by the initial infected traveler and all their offspring must equate with the expected value that these N_0 chains become extinct, so that

$$q_0 \;=\; \text{E}\left[q^{N_0}\right] \;=\; \text{E}\left[q^{Y+\sum_{i=1}^{X} Z_i}\right].$$

Consider a single, recently-infected traveler from the source region. Let

T = time since infection at scheduled departure;

A_D = event "traveler avoids detection when screened at departure (at time T)";

X = number of in-flight infections by this traveler;

σ = sensitivity of symptomatic screening upon arrival;

A_A = event "traveler avoids detection when screened at arrival (at time $T + d_F$)";

Y = number of infections by this traveler after entering the community.

We want

$$\mathrm{E}\left[q^{Y+\sum_1^X Z_i}\right].$$

As A_D, X, A_A and Y are most easily determined when we know T, we begin by conditioning on $T = t$. Note that

$$\mathrm{E}\left[q^{Y+\sum_1^X Z_i}\right] \;=\; \int_0^{10} \mathrm{E}\left[q^{Y+\sum_1^X Z_i} \,\middle|\, T = t\right] f_T(t)\mathrm{d}t\,.$$

We then write

$$\begin{aligned}
&\mathrm{E}\left[q^{Y+\sum_1^X Z_i} \,\middle|\, T = t\right] \\
&= \mathrm{E}\left[q^{Y+\sum_1^X Z_i} \,\middle|\, T = t, A_D\right] \Pr\left(A_D \,|\, T = t\right) \;+\; \mathrm{E}\left[q^{Y+\sum_1^X Z_i} \,\middle|\, T = t, \overline{A}_D\right] \Pr\left(\overline{A}_D \,|\, T = t\right) \\
&= \mathrm{E}\left[q^{Y+\sum_1^X Z_i} \,\middle|\, T = t, A_D\right] \Pr\left(A_D \,|\, T = t\right) \;+\; \Pr\left(\overline{A}_D \,|\, T = t\right)\,,
\end{aligned}$$

where

$$\Pr(\overline{A}_D \,|\, T = t) \;=\; 1 - \Pr(A_D \,|\, T = t) \;=\; \begin{cases} 0, & \text{if } 0 \le t \le 2, \\ \sigma, & \text{if } 2 < t \le 10. \end{cases}$$

To get $\mathrm{E}\left[q^{Y+\sum_1^X Z_i} \,\middle|\, T = t, A_D\right]$ we do some more conditioning. First

$$\mathrm{E}\left[q^{Y+\sum_1^X Z_i} \,\middle|\, T = t, A_D\right] = \sum_x \mathrm{E}\left[q^{Y+\sum_1^X Z_i} \,\middle|\, T = t, A_D, X = x\right] \Pr(X = x \mid T = t, A_D)$$

and then

$$\begin{aligned}
&\mathrm{E}\left[q^{Y+\sum_1^X Z_i} \,\middle|\, T = t, A_D, X = x\right] \\
&\qquad = \mathrm{E}\left[q^{Y+\sum_1^X Z_i} \,\middle|\, T = t, A_D, X = x, A_A\right] \Pr(A_A \,|\, T = t, A_D, X = x) \\
&\qquad\quad + \mathrm{E}\left[q^{Y+\sum_1^X Z_i} \,\middle|\, T = t, A_D, X = x, \overline{A}_A\right] \Pr(\overline{A}_A \,|\, T = t, A_D, X = x),
\end{aligned}$$

where

$$\Pr(\overline{A}_A \,|\, T = t, A_D, X = x) \;=\; 1 - \Pr(A_A \,|\, T = t, A_D, X = x) \;=\; \begin{cases} 0, & \text{if } 0 \le t + d_F \le 2, \\ \sigma, & \text{if } 2 < t + d_F \le 10, \end{cases}$$

$$\mathrm{E}\left[q^{Y+\sum_1^X Z_i} \,\middle|\, T = t, A_D, X = x, A_A\right]$$

$$
\begin{aligned}
&= \mathrm{E}\left[q^Y \,\middle|\, T=t, A_D, X=x, A_A\right] \; \mathrm{E}\left[q^{\sum_1^X Z_i} \,\middle|\, T=t, A_D, X=x, A_A\right] \\
&= \mathrm{E}\left[q^Y \,\middle|\, T=t, A_D, A_A\right] \; \mathrm{E}\left[q^{\sum_1^X Z_i} \,\middle|\, X=x\right] \\
&= \exp\left[\int_{t+d_F}^{t+d_F+\delta} \beta(u)\mathrm{d}u\,(q-1)\right] \exp[R(q-1)x] \\
&= \exp\left[\int_{t+d_F}^{t+d_F+\delta} \beta(u)\mathrm{d}u\,(q-1)\right] q^x
\end{aligned}
$$

and

$$
\mathrm{E}\left[q^{Y+\sum_1^X Z_i} \,\middle|\, T=t, A_D, X=x, \overline{A}_A\right] \;=\; \mathrm{E}\left[q^{\sum_1^X Z_i} \,\middle|\, X=x\right] \;=\; \exp[R(q-1)x] \;=\; q^x.
$$

Using the fact that $\Pr(A_A \mid T=t, A_D, X=x) = \Pr(A_A \mid T=t, A_D)$, i.e. event A_A does not depend on X for this control measure, we find

$$
\begin{aligned}
\mathrm{E}\left[q^{Y+\sum_1^X Z_i} \,\middle|\, T=t, A_D\right] \;=\; & \Pr(A_A \mid T=t, A_D)\, \mathrm{E}\left[q^X \,\middle|\, T=t, A_D\right] \\
& \times \exp\left[\int_{t+d_F}^{t+d_F+\delta} \beta(u)\mathrm{d}u\,(q-1)\right] \\
& + \Pr(\overline{A}_A \mid T=t, A_D)\, \mathrm{E}\left[q^X \,\middle|\, T=t, A_D\right].
\end{aligned}
$$

Finally, as

$$
X \,\Big|\, T=t \text{ and } A_D \quad \sim \quad \text{Poisson}\left(\lambda \int_t^{t+d_F} \beta(u)\mathrm{d}u\right),
$$

$\mathrm{E}\left[q^X \,\middle|\, T=t, A_D\right] \;=\; \exp\left[\lambda \int_t^{t+d_F} \beta(u)\mathrm{d}u\ (q-1])\right].$

Calculating the daily probability of an epidemic being initiated

On any one day, the probability that an epidemic is initiated (p) is determined by the number of infected travelers attempting to travel from the source region on that day (K), the probability they evade detection during departure screening in the source region (θ_D), the probability that the evade detection during arrival screening in the at-risk country conditional on having evaded screening on departure (θ_A), and the probability they and their offspring fail to initiate an epidemic (q_0). Let n be the number of travelers intending to depart the source region and π be the prevalence of infected individuals in the source country on the day. As this prevalence is expected to be low, at least initially, the probability distribution for the number of infected travelers (K) attempting to depart from the source region on the day can be approximated as Poisson with mean $n\pi$.

When only cases detected by screening are removed at departure and arrival, the probability ($\bar{p}$) that an epidemic is not initiated is

$$
\bar{p} \;=\; \mathrm{E}\Big[{q_0}^k\Big] \;=\; \exp\Big(-n\pi(1-q_0)\Big).
$$

When border control at arrival consist of quarantining an entire flight when a case is detected by screening, some further calculations are needed. Let

E = event "Infected travelers departing on day fail to initiate an epidemic";

D_A = event "At least one infected traveler on day is detected on arrival";

K_D = number of infected travelers successfully departing on day.

The probability of an epidemic not being initiated on day is given by:

$$\overline{p} \;=\; \Pr(E) \;=\; \sum_k \Pr(E|K_D = k)\,\Pr(K_D = k).$$

Note that

$$\begin{aligned}\Pr\left(E\middle|K_D = k\right) & \\ &= \Pr(E\big|K_D = k, D_A)\,\Pr(D_A\big|K_D = k) \;+\; \Pr(E\big|K_D = k, \overline{D}_A)\,\Pr(\overline{D}_A\big|K_D = k) \\ &= \;1(1-\theta_A^k) \;+\; q_0^k\theta_A^k,\end{aligned}$$

so

$$\overline{p} \;=\; \sum_k \left(1 - \theta_A^k + (q_0\theta_A)^k\right)\Pr(K_D = k).$$

As K_D is distributed as Poisson with mean $n\pi\theta_D$, standard probability generating function results give

$$\overline{p} \;=\; 1 \;-\; \exp\left(-\,n\pi\theta_D(1-\theta_A)\right) \;+\; \exp\left(-\,n\pi\theta_D(1-q_0\theta_A)\right).$$
